# Synaptic Remodeling in the Cone Pathway After Early Postnatal Horizontal Cell Ablation

**DOI:** 10.3389/fncel.2021.657594

**Published:** 2021-05-26

**Authors:** Lena Nemitz, Karin Dedek, Ulrike Janssen-Bienhold

**Affiliations:** ^1^Visual Neuroscience, Department of Neuroscience, University of Oldenburg, Oldenburg, Germany; ^2^Animal Navigation/Neurosensorics, Institute for Biology and Environmental Sciences, University of Oldenburg, Oldenburg, Germany; ^3^Research Center Neurosensory Science, University of Oldenburg, Oldenburg, Germany

**Keywords:** bipolar cells, horizontal cells, photoreceptors, cones, ribbon synapse, synaptic remodeling, retina, vision

## Abstract

The first synapse of the visual pathway is formed by photoreceptors, horizontal cells and bipolar cells. While ON bipolar cells invaginate into the photoreceptor terminal and form synaptic triads together with invaginating horizontal cell processes, OFF bipolar cells make flat contacts at the base of the terminal. When horizontal cells are ablated during retina development, no invaginating synapses are formed in rod photoreceptors. However, how cone photoreceptors and their synaptic connections with bipolar cells react to this insult, is unclear so far. To answer this question, we specifically ablated horizontal cells from the developing mouse retina. Following ablation around postnatal day 4 (P4)/P5, cones initially exhibited a normal morphology and formed flat contacts with OFF bipolar cells, but only few invaginating contacts with ON bipolar cells. From P15 on, synaptic remodeling became obvious with clustering of cone terminals and mislocalized cone somata in the OPL. Adult cones (P56) finally displayed highly branched axons with numerous terminals which contained ribbons and vesicular glutamate transporters. Furthermore, type 3a, 3b, and 4 OFF bipolar cell dendrites sprouted into the outer nuclear layer and even expressed glutamate receptors at the base of newly formed cone terminals. These results indicate that cones may be able to form new synapses with OFF bipolar cells in adult mice. In contrast, cone terminals lost their invaginating contacts with ON bipolar cells, highlighting the importance of horizontal cells for synapse maintenance. Taken together, our data demonstrate that early postnatal horizontal cell ablation leads to differential remodeling in the cone pathway: whereas synapses between cones and ON bipolar cells were lost, new putative synapses were established between cones and OFF bipolar cells. These results suggest that synapse formation and maintenance are regulated very differently between flat and invaginating contacts at cone terminals.

## Introduction

At the visual system’s first synapse, cone photoreceptors provide synaptic input to horizontal cells, ON bipolar cells and OFF bipolar cells, thereby splitting the light information into parallel pathways. Two different types of contacts can be distinguished at the cone terminal: Invaginating contacts with horizontal and ON bipolar cells (triads) and flat contacts with OFF bipolar cells (Haverkamp et al., 2000). Previous electron microscopic studies have revealed the chronological sequence of cone synaptogenesis in the mouse retina (Olney, 1968; Blanks et al., 1974; Rich et al., 1997; Sherry et al., 2003). When cone terminals begin to invade the outer plexiform layer (OPL) at postnatal day 4 (P4)/P5, the ribbon, a specialized structure that binds synaptic vesicles and enables a rapid and graded release of glutamate, is attached to the membrane and the cone terminal makes a contact with one horizontal cell dendrite. Starting at P6, this dendrite invaginates into the cone pedicle together with a second horizontal cell dendrite and both horizontal cell processes are positioned lateral to the ribbon. Between P7 and P10, triads are completed by the addition of one or two ON bipolar cell dendrites that occupy the central position below the synaptic ribbon.

Although the chronological order of cone synapse formation is well-described, the molecular mechanisms that underlie this process are not fully understood. Recently, the dystroglycan-pikachurin complex of photoreceptors has been reported to interact transsynaptically with GPR179, a G protein-coupled receptor that is expressed at the dendritic tips of ON bipolar cells (Orlandi et al., 2018). The knock-out of dystroglycan (Satz et al., 2009; Omori et al., 2012) and pikachurin (Sato et al., 2008) leads to improper photoreceptor synapse formation and visual impairment. Moreover, an ablation of horizontal cells from the adult retina results in a loss of synaptic contacts between photoreceptors and ON bipolar cells (Sonntag et al., 2012; Keeley et al., 2013; Wu et al., 2013) and an elimination of horizontal cells during early postnatal development impedes the invagination of rod bipolar cells into rod terminals (Nemitz et al., 2019). However, it remains unclear how cones and cone bipolar cells react when horizontal cells are ablated during development.

In the present study, we analyzed the effects of an early postnatal loss of horizontal cells on the morphology of cone photoreceptors and their synaptic contacts with bipolar cells. For this purpose, horizontal cells were specifically ablated from the mouse retina around P4/P5 (at the time when horizontal cells start to make the first contacts with cone pedicles) using diphtheria toxin receptor (DTR)-mediated cell knock-out. Our immunohistochemical and electron microscopic analysis showed that cones initially displayed a normal morphology and established flat contacts with OFF bipolar cells, but only few invaginating synapses with ON bipolar cells. Beginning at P15, cone somata were partly mislocalized and cone terminals clustered in the OPL and lost all invaginating ON bipolar cell invaginations. In the adult retina, cones and their postsynaptic partners underwent even more severe structural changes, including an aberrant cone neurite sprouting and a formation of new putative synapses between cones and OFF bipolar cells. These findings demonstrate the potential of the adult retina for morphological plasticity and highlight again the importance of horizontal cells for the maintenance of invaginating synapses (Sonntag et al., 2012).

## Materials and Methods

### Animals

Cx57-DTRfrtCre mice which express the primate DTR (kind gift from Dr. T. Buch, TU München, Munich, Germany) under the control of the endogenous connexin57 (Cx57) promoter have been previously described (Sonntag et al., 2012) and can be obtained from the European Mouse Mutant Archive (EM:06024). Mice were backcrossed into the C57BL/6J background for at least three generations. Animals were maintained under a 12 h light/dark cycle with food and water *ad libitum.* Mice of both sexes were used in the experiments. All procedures were approved by the local animal welfare committee (*Niedersächsisches Landesamt für Verbraucherschutz und Lebensmittelsicherheit*, Az:33.19-542502-04-12/0995) and were in accordance with the law on animal protection issued by the German Federal Government (*Tierschutzgesetz*).

### DT Injections

Cx57^+/DTR^ and Cx57^+/+^ (control) littermates were injected intraperitoneally with 12.5–20 ng DT (Sigma) at P4 and P5.

### Tissue Preparation

Tissue preparation was done as previously described in Nemitz et al. (2019). Briefly, mice were killed by decapitation (P8 and P11) or anesthetized with CO_2_ and killed by cervical dislocation (P15, P21, and P56). Eyes were enucleated, transferred into physiological phosphate buffer saline (pH 7.4) and cornea, lens and vitreous body were removed. For immunohistochemistry of retinal cryosections, posterior eyecups were fixed in 2% paraformaldehyde (PFA) and 3% sucrose in 0.1 M phosphate buffer (PB, pH 7.4) for 20 min and washed in 0.1 M PB. After cryoprotection in 30% sucrose in 0.1 M PB overnight at 4°C, eyecups were embedded in Tissue-Tek O.C.T. Compound (Sakura Finetek) and cut into vertical sections (20 μm) using a cryostat (Leica CM1860). For immunohistochemistry of whole mounts, retinae were isolated from the eyecups, mounted on filter paper ganglion cell side up and fixed with 2% PFA and 3% sucrose in 0.1 M PB for 20 min. After washing in 0.1 M PB, retinae were cryoprotected in 30% sucrose in 0.1 M PB overnight at 4°C and subjected to three freeze-thaw cycles. For electron microscopy, isolated retinae were fixed in 1% PFA, 3% sucrose and 2.5% glutaraldehyde in 0.05 M PB overnight at 4°C, washed in 0.1 M PB and post-fixed with 1% OsO_4_ in 0.1 M PB for 1 h.

### Immunohistochemistry and Image Acquisition

Immunostainings were performed as described earlier (Nemitz et al., 2019). Cryosections and whole mounts of *Cx57*^+/+^ (*n* = 3–6) and *Cx57*^+/DTR^ (*n* = 3–6) mice were washed in 0.1 M PB and blocked with 5% ChemiBLOCKER (Millipore), 0.3% Triton X-100 and 0.02% NaN_3_ in 0.1 M PB for 1 h at room temperature (cryosections) or overnight at 4°C (whole mounts). Primary antibodies (Table 1) were diluted in blocking solution and applied overnight (cyrosections) or for 5 days (whole mounts) at 4°C. After washing in 0.1 M PB, tissue was incubated with secondary antibodies (conjugated to Alexa 488, Alexa 588 or Alexa 647, Thermo Fisher Scientific, 1:600) in blocking solution for 2 h at room temperature (cryosections) or 2 days (whole mounts) at 4°C. Finally, cryosections and whole mounts were washed in 0.1 M PB and mounted in Vectashield (Vector Laboratories).

**TABLE 1:** List of primary antibodies used in this study.

Antibody	Host, type	Dilution	Source, Catalog #, RRID
Calbindin D-28k	Rabbit, polyclonal	1:500	Swant, CB-38, AB_2721225
Cone arrestin	Rabbit, polyclonal	1:1,000	Millipore, AB15282, AB_1163387
Calsenilin	Mouse, monoclonal	1:2,000	Millipore, 05-756, AB_309969
CtBP2	Mouse, monoclonal	1:5,000	BD Biosciences, 612044, AB_399431
Ca_*v*_1.1	Mouse, monoclonal	1:500	Millipore, MAB427, AB_2069582
GluK1	Mouse, monoclonal	1:200	Santa Cruz Biotechnology, sc-393420, AB_2716684
HCN4	Rat, polyclonal	1:100	Gift from Frank Müller (FZ Jülich, Jülich, Germany)
PKARIIβ	Mouse, monoclonal	1:1,500	BD Biosciences, 610625, AB_397957
SCGN	Sheep, polyclonal	1:1,000	BioVendor Laboratory Medicine, RD184120100, AB_2034062
VGluT1	Guinea pig, polyclonal	1:2,000	Millipore, AB5905, AB_2301751

Images were acquired using a confocal laser scanning microscope (Leica TCS SP8). Retinal sections were scanned with an HC PL APO CS2 63x/1.4 oil objective. Whole mounts were scanned with an HC PL APO CS2 40x/1.3 oil objective. Maximum projections of confocal stacks (0.2 μm single scans) are shown. Brightness and contrast were adjusted for presentation purposes using Fiji (Schindelin et al., 2012).

### Electron Microscopy

As earlier described (Nemitz et al., 2019), fixed retinae of *Cx57*^+/+^ (*n* = 2–4) and *Cx57*^+/DTR^ mice (*n* = 3–5) were washed in 0.1 M PB and dehydrated in increasing acetone concentrations (50–100%). After embedding in Agar 100 Resin (Agar Scientific), retinae were sectioned vertically (90 nm) using a Reichert-Jung Ultracut E ultramicrotome. Ultrathin sections were collected on copper grids and examined with a Zeiss EM 902A electron microscope. Brightness and contrast of electron micrographs were adjusted in Adobe Photoshop CS6 Extended (Adobe Systems).

### Quantification and Statistical Analysis

For the quantification of cones, we counted the number of cone somata in cone arrestin-stained vertical sections of *Cx57*^+/+^ (*n* = 3) and *Cx57*^+/DTR^ (*n* = 3) mice. For each animal, 12 images (198.39 × 198.39 μm) were analyzed. Cones that had their soma within the distal 50% of the ONL were categorized as correctly positioned cones and cones that had their soma within the proximal 50% of the ONL, the INL or the OPL were categorized as mislocalized cones.

Data were analyzed in GraphPad Prism 5 (GraphPad Software). Numbers of cones were normally distributed (D’Agostino-Pearson normality test). Therefore, differences between genotypes were tested for statistical significance using an unpaired *t*-test. To compare the distribution of cone somata in wild-type and horizontal cell-ablated mice, Fisher’s exact test was used. Values are given as mean ± standard deviation (SD).

## Results

### Ablation of Horizontal Cells

**FIGURE 1:**
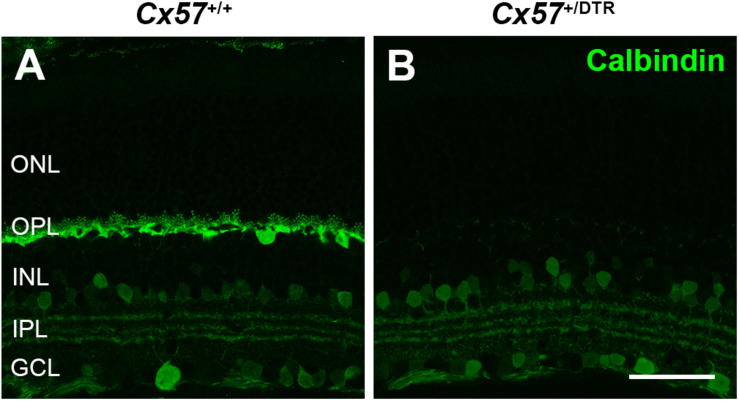
Horizontal cell ablation. **(A,B)** Retinal cryosections of *Cx57*^+/+^ and *Cx57*^+/DTR^ mice (P56) were labeled with an antibody specific for the horizontal cell marker calbindin. In *Cx57*^+/DTR^ mice, horizontal cells were completely lost, while calbindin-positive amacrine and ganglion cells were unaffected **(B)**. Scale bar, 50 μm.

To investigate how cones and their postsynaptic partners respond to a loss of horizontal cells during early postnatal development, we ablated horizontal cells via DTR-mediated cell knock-out (Saito et al., 2001). To this end, we used *Cx57*^+/DTR^ mice that selectively express the DTR from primates selectively in horizontal cells (Sonntag et al., 2012) and induced an apoptosis of horizontal cells by the injection of DT at P4 and P5 (Nemitz et al., 2019). At that time, cone synaptogenesis has just started and cone terminals begin to form the first contacts with horizontal cells (Sherry et al., 2003). To confirm the ablation of horizontal cells, vertical sections of *Cx57*^+/+^ and *Cx57*^+/DTR^ retinae were stained for calbindin, a marker for horizontal cells. Calbindin-positive horizontal cells were present in DT-injected *Cx57*^+/+^ mice (Figure 1A) and absent in DT-injected *Cx57*^+/DTR^ mice (Figure 1B). In contrast, calbindin-immunoreactive inner retinal neurons were comparable in both genotypes (Figures 1A,B).

### Cone Neurite Sprouting After Early Postnatal Horizontal Cell Ablation

**FIGURE 2:**
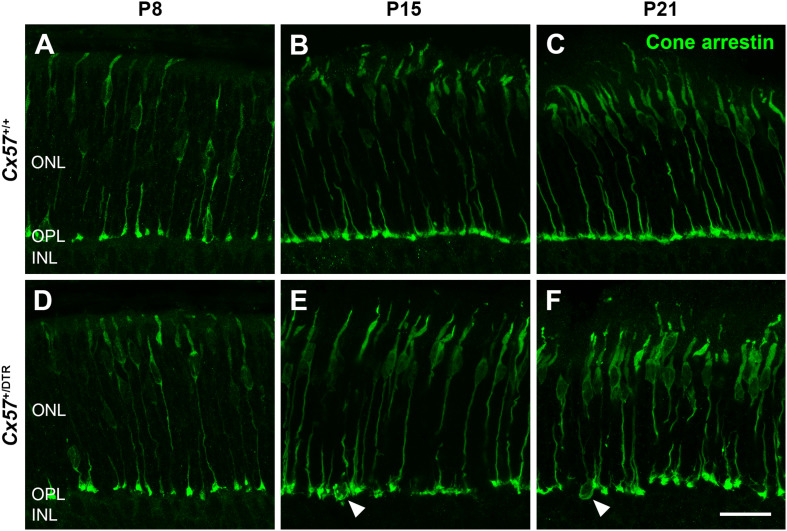
Mislocalized cone somata following early postnatal horizontal cell ablation. **(A–F)** Retinae of *Cx57*^+/+^ and *Cx57*^+/DTR^ mice of different ages (P8, P15, and P21) were stained for cone arrestin, a marker for cone photoreceptors. In wild-type mice, cones displayed the classical morphology including outer segment, inner segment, cell body, axon and synaptic terminal **(A–C)**. Cone somata were scattered across the entire ONL at P8 **(A)** and positioned in the distal part of the ONL from P15 to P21 **(B,C)**. In *Cx57*^+/DTR^ mice, the cone morphology was initially comparable to that in wild-type mice **(A,D)**. From P15 onward, cone somata were partly mislocalized (arrowheads) **(E,F)** and cone terminals were irregularly distributed in the OPL **(E,F)**. Scale bar, 50 μm.

**FIGURE 3:**
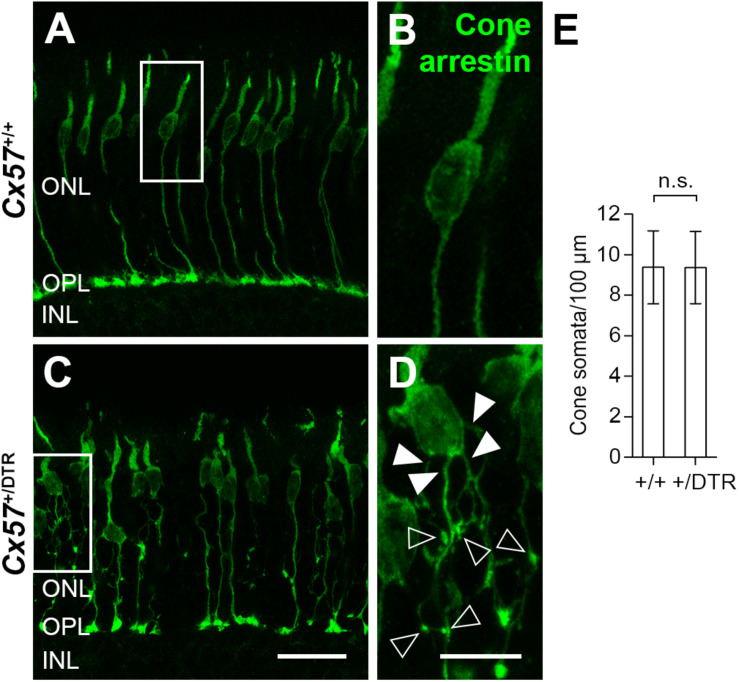
Cone neurite sprouting in adult horizontal cell-ablated mice. **(A–D)** Vertical sections of *Cx57*^+/+^ and *Cx57*^+/DTR^ mice (P56) were labeled with antibodies against cone arrestin. While cones in wild-type mice had one axon with a single terminal **(A,B)**, cones in horizontal cell-ablated mice had branched axons with numerous terminals (open arrowheads) **(C,D)**. In some cases, several axons emerged from one soma (white arrowheads) **(C,D)**. **(E)** Quantification of cone somata in vertical sections of wild-type (*n* = 3) and horizontal cell-ablated retinae (*n* = 3) (P56). *p* = 0.9736, *t*-test. Values are presented as mean ± SD. Scale bars, 25 μm **(C)**, 10 μm **(D)**.

**FIGURE 4:**
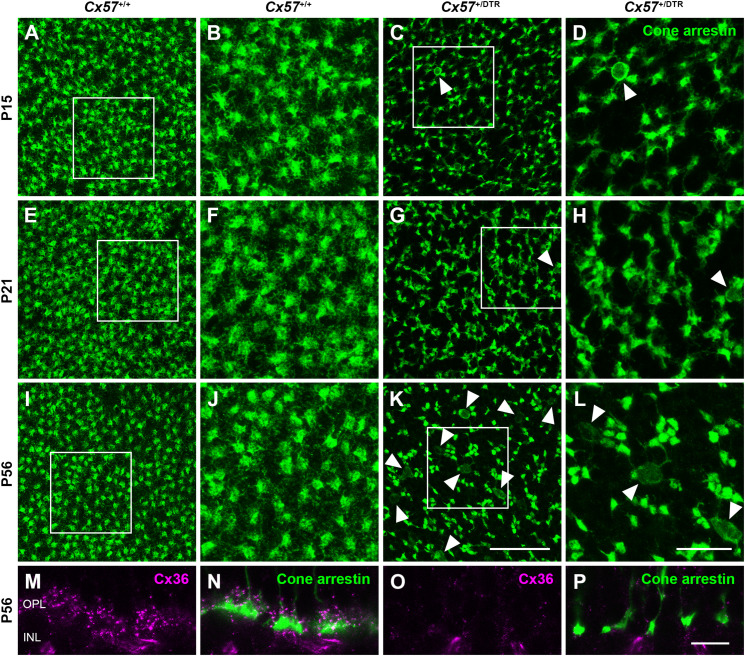
Clustering and reduced gap junctional coupling of cone terminals in horizontal cell-ablated mice. **(A–L)** Immunolabeling of retinal whole mounts from *Cx57*^+/+^ and *Cx57*^+/DTR^ mice with antibodies against cone arrestin. In the OPL of wild-type mice, cone pedicles formed a regular mosaic **(A,B,E,F,I,J)**, whereas in horizontal cell-ablated mice, cone pedicles were unevenly spaced and several mislocalized cone somata were apparent (arrowheads) **(C,D,G,H,K,L)**. **(M–P)** Vertical sections of *Cx57*^+/+^ and *Cx57*^+/DTR^ retinae were stained for cone arrestin (green) and Cx36 (magenta). Cx36 expression in the OPL of horizontal cell-ablated mice was strongly reduced compared to wild-type mice. Scale bars, 25 μm **(K)**, 10 μm **(L,P)**.

To monitor the development of cone morphology after horizontal cell ablation, we stained retinal cryosections of *Cx57*^+/+^ and *Cx57*^+/DTR^ mice (P8, P15, and P21) with an antibody against cone arrestin. In wild-type mice, cones showed the typical morphology composed of an outer and inner segment, a soma and an axon with a synaptic terminal (Figures 2A–C). During the second postnatal week, cone photoreceptors undergo a phase of translocation before they occupy their final position in the distal ONL (Rich et al., 1997). Accordingly, cone somata were distributed over the entire ONL width in *Cx57*^+/+^ mice at P8 (Figure 2A) but located in the outermost part of the ONL from P15 onward (Figures 2A–C). At P8, the morphology of cones and the distribution of cone somata in horizontal cell-ablated mice was undistinguishable from that in control mice (Figures 2A,D). Beginning at P15, however, we found mislocalized cone somata at the level of the OPL (Figures 2E,F, arrowheads). Furthermore, cone terminals appeared to be irregularly spaced and seemed to start forming clusters (Figures 2E,F). At P56, differences in the morphology of cones in *Cx57*^+/+^ and *Cx57*^+/DTR^ mice became even more evident (Figures 3A–D). Occasionally, two or more axons emerging from one soma were observed (Figure 3D, white arrowheads). Moreover, axons showed branching into several collaterals with small and often more slender appearing terminals (Figure 3D, open arrowheads). The appearance of several terminals in one cone cell at P56 suggests that cones formed new terminals in adult mice. Despite these morphological changes, the number of cones did not differ between the two genotypes at P56 (Figure 3E; *Cx57*^+/+^: 9.38 ± 1.80 cone somata per 100 μm; mean ± SD; *Cx57*^+/DTR^: 9.37 ± 1.78 cone somata per 100 μm, *p* = 0.9736, *t*-test), indicating that cones did not undergo cell death. In contrast to the number of cones, the distribution of cone somata across the depth of the ONL was significantly different between *Cx57*^+/+^ and *Cx57*^+/DTR^ mice at P56 (*p* = 0.0309, Fisher’s exact test). In wild-type mice, all analyzed cone somata (670/670) were localized in the distal ONL, whereas in horizontal cell-ablated mice, 99.3% (664/669) of the cone somata were correctly positioned and 0.7% (5/669) of the cone somata were found mispositioned in the proximal ONL, distal INL, or OPL. Immunolabeling of retinal whole mounts for cone arrestin confirmed that the regular mosaic of cone terminals found at P15, P21 and P56 in the OPL of control mice (Figures 4A,B,E,F,I–L) was absent in age-matched *Cx57*^+/DTR^ mice. Instead, cone pedicles were unevenly distributed and formed clusters (Figures 4C,D,G,H,K,L). In addition, cone terminals appeared smaller and the telodendrial network seemed to be reduced (Figures 4C,D,G,H,K,L). Consistently, the immunoreactivity for the gap junction protein Connexin36 (Cx36) in the OPL of horizontal cell-ablated mice was strongly decreased (Figures 4M–P), suggesting that gap junctional coupling between photoreceptors is disrupted. In addition to that, peanut agglutinin (PNA) labeling in the OPL was considerably reduced, which indicates that the extracellular matrix at cone terminals is altered in Cx57^+/DTR^ retinae (Supplementary Figure 1).

### New Cone Terminals Contain Ribbons and Vesicular Glutamate Transporters

**FIGURE 5:**
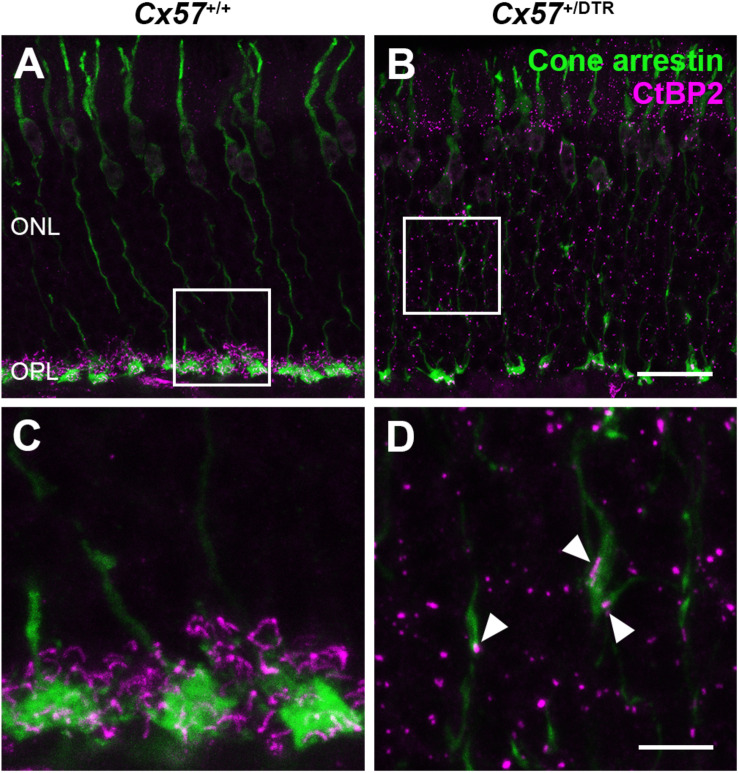
Ribbons in new cone terminals. **(A–D)** Double staining of vertical sections from *Cx57*^+/+^ and *Cx57*^+/DTR^ mice (P56) for cone arrestin, a cone marker (green), and CtBP2 (magenta), a synaptic ribbon marker. In the outer retina of wild-type mice, ribbons were horseshoe shaped and confined to the OPL **(A,C)**. By contrast, ribbons in horizontal cell-ablated mice were smaller and distributed over the entire ONL **(B,D)**. In addition, CtBP2-postive structures were frequently found in ectopic cone terminals (arrowheads) **(D)**. Scale bars, 20 μm **(B)**, 5 μm **(D)**.

**FIGURE 6:**
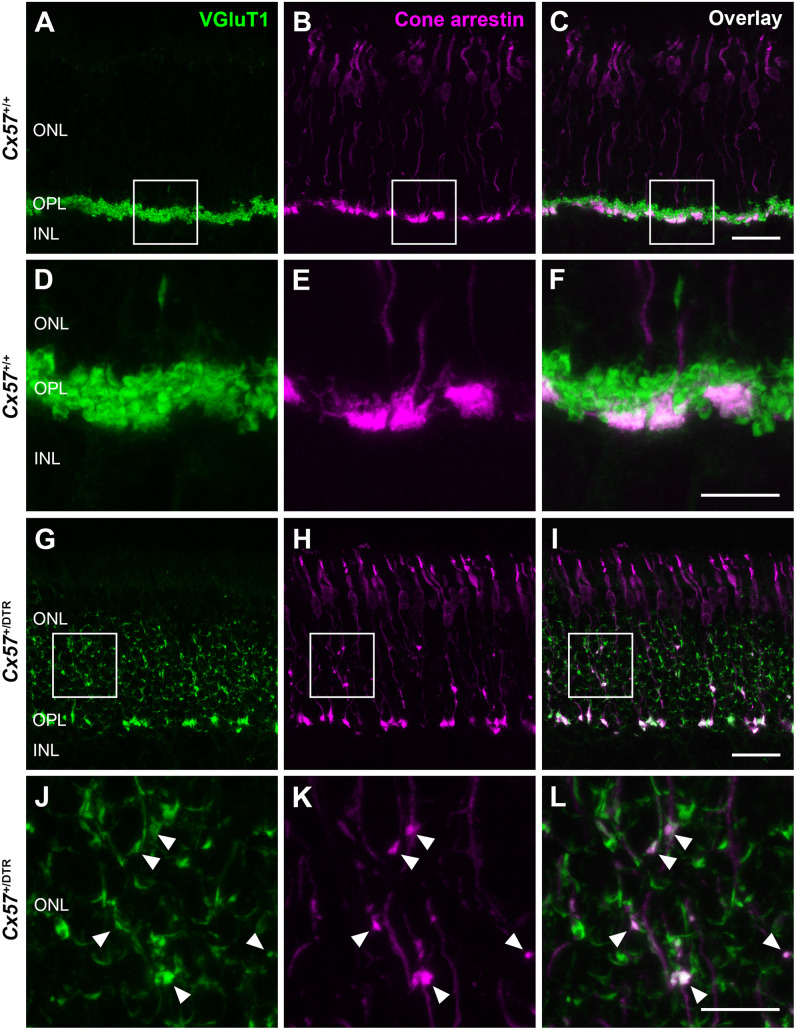
New cone terminals express vesicular glutamate transporters. **(A–L)** Retinal cryosections of *Cx57*^+/+^ and *Cx57*^+/DTR^ mice (P56) were stained for the vesicular glutamate transporter VGluT1 (green) and cone arrestin (magenta). In wild-type and horizontal cell-ablated mice, cone pedicles in the OPL were VGluT1-positive **(A–I)**. Moreover, VGluT1 immunoreactivity was present within ectopic cone terminals in the ONL of *Cx57*^+/DTR^ mice (arrowheads) **(J,K,L)**. Scale bars, 25 μm **(C,I)**, 10 μm **(F,L)**.

To check whether the newly formed cone terminals in adult *Cx57*^+/DTR^ mice (P56) have synaptic ribbons, we labeled cones with antibodies specific for cone arrestin and ribbons with antibodies specific for CtBP2, a protein that is identical to the B domain of the ribbon component RIBEYE (Schmitz et al., 2000). Ribbons in the outer retina of control mice displayed the classical horseshoe shape and were restricted to the OPL (Figures 5A,C). By contrast, ribbons of *Cx57*^+/DTR^ mice were shorter as evidenced by a punctate CtBP2 labeling and not only present in the OPL, but predominately distributed throughout the entire ONL (Figures 5B,D). Furthermore, the double labeling revealed that ribbons were often present within slender ectopic cone terminals in the ONL (Figure 5D, arrowheads). In addition to that, we stained retinae from wild-type and horizontal cell-ablated mice (P56) for cone arrestin and vesicular glutamate transporter 1 (VGluT1), which is responsible for the uptake of glutamate into synaptic vesicles and expressed in photoreceptor terminals (Johnson et al., 2003; Sherry et al., 2003; Figures 6A–F). In *Cx57*^+/+^ and *Cx57*^+/DTR^ mice, vGluT1 immunoreactivity was present in cone pedicles in the OPL (Figures 6A–I). Similar to the CtBP2 labeling, VGluT1 labeling was also found in ectopic cone terminals in the ONL (Figures 6J–L, arrowheads). Taken together, these findings demonstrate that newly formed cone terminals possess synaptic elements that are critical for glutamate release of photoreceptors.

### Cone Terminals Lose Invaginations From ON Bipolar Cells

**FIGURE 7:**
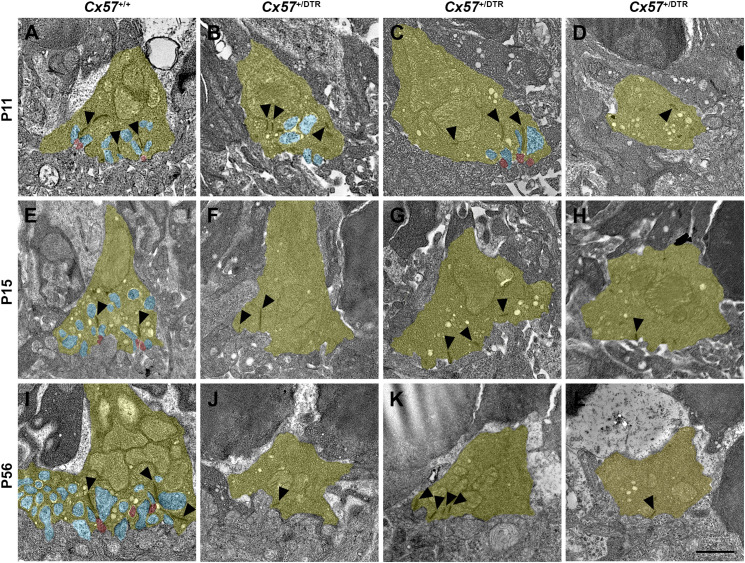
Loss of ON bipolar cell invaginations in cone terminals. **(A–L)** Electron microscopic analysis of individual cone terminals from *Cx57*^+/+^ and *Cx57*^+/DTR^ mice at different ages (P11, P15, and P56). Cone terminals (yellow) in wild-type mice contained triads composed of horizontal cell dendrites (blue) and ON bipolar cell dendrites (red) as early as P11 **(A,E,I)**. In horizontal cell-ablated mice, some cone terminals with horizontal cell invaginations were found at P11 **(B)**. However, triads were rarely observed **(C)** and most cone pedicles contained no invaginations **(D)**. At P15 and P56, cone terminals were generally lacking any invaginations in *Cx57*^+/DTR^ mice **(F–H,J–L)**. Ribbons were often shorter and free-floating (arrowheads) **(B–D,F–H,J–L)**. Scale bar, 1 μm.

For the examination of synapses between cones and ON bipolar cells we analyzed cone terminals from wild-type and horizontal cell-ablated mice of different ages (P11, P15, and P56) by electron microscopy. In *Cx57*^+/+^ mice, triads comprising horizontal and ON bipolar cell dendrites were present at all analyzed time points (Figures 7A,E,I). Although horizontal cell ablation was already induced at P4/P5, we occasionally found remnants from horizontal cell invaginations in *Cx57*^+/DTR^ mice at P11 (Figure 7B). However, complete triads were rare (Figure 7C) and most of the cone terminals revealed a complete lack of invaginations (Figure 7D), suggesting that horizontal cells may not only be essential for the invagination of rod bipolar cells into rod terminals (Nemitz et al., 2019), but also for the invagination of cone ON bipolar cells into cone terminals. At P15 and P56, cone pedicles of horizontal cell-ablated mice did not show any invaginations (Figures 7F–H,J–L), indicating that cones have lost the remaining contacts with ON bipolar cells. In line with the CtBP2 labeling (Figures 5B,D), synaptic ribbons in *Cx57*^+/DTR^ mice were often shorter and not anchored to the cell membrane (Figures 7B–D,F-H,J–L, arrowheads). Unfortunately, we were not able to unambiguously identify newly formed cone terminals in electron micrographs since ectopic rod terminals are also present in the ONL of horizontal cell-ablated mice (Nemitz et al., 2019) and common criteria for the differentiation between rod and cone terminals such as the terminal size or the number of mitochondria, ribbons and invaginations were not applicable for ectopic rod and cone terminals in horizontal cell-ablated mice.

**FIGURE 8:**
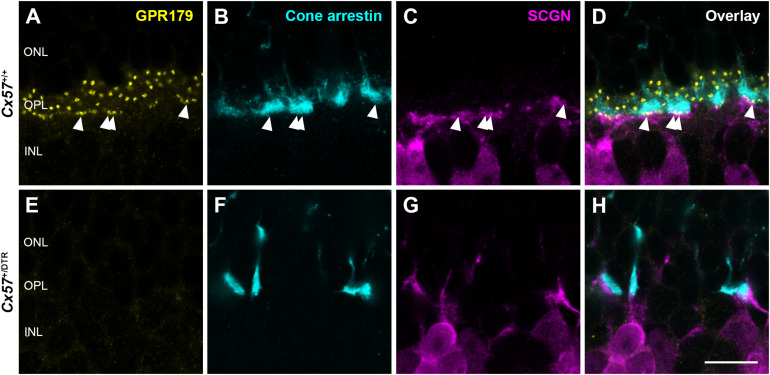
Absence of functional synapses between cones and ON bipolar cells in adult horizontal cell-ablated mice. **(A–H)** Triple labeling of retinae from *Cx57*^+/+^ and *Cx57*^+/DTR^ mice (P56) for GPR179 (a component of the mGluR6 macromolecular complex, yellow), cone arrestin (a marker for cones, blue) and SCGN (a marker for a subset of ON and OFF bipolar cells, magenta). In wild-type mice, GPR179-immunoreactive puncta were observed at contact points between cone pedicles and bipolar cell dendrites (arrowheads) **(A–D)**. In contrast, GPR179 labeling was completely absent in *Cx57*^+/DTR^ mice **(E–H)**, suggesting that adult horizontal cell-ablated mice lack functional synapses between cones and ON bipolar cells. Scale bar, 10 μm.

To confirm the absence of synaptic contacts between cones and ON bipolar cells in adult *Cx57*^+/DTR^ mice (P56), we stained the mGluR6 signaling complex with an antibody against Ca_*v*_1.1, that cross-reacts with GPR179 (Hasan et al., 2016). Additionally, we labeled cones with an antibody against cone arrestin and a subset of cone bipolar cells with an antibody against secretagogin (SCGN, Puthussery et al., 2010). While GPR179-positive puncta were found at contact points between cone terminals and secretagogin-immunoreactive bipolar cells in *Cx57*^+/+^ mice (Figures 8A–D, arrowheads), GPR179 labeling was completely missing in *Cx57*^+/DTR^ mice (Figures 8E–H), suggesting that functional synapses between cones and ON bipolar cells are indeed absent in adult horizontal cell ablated mice.

### Sprouting OFF Bipolar Cell Dendrites Form New Contacts With Cones

**FIGURE 9:**
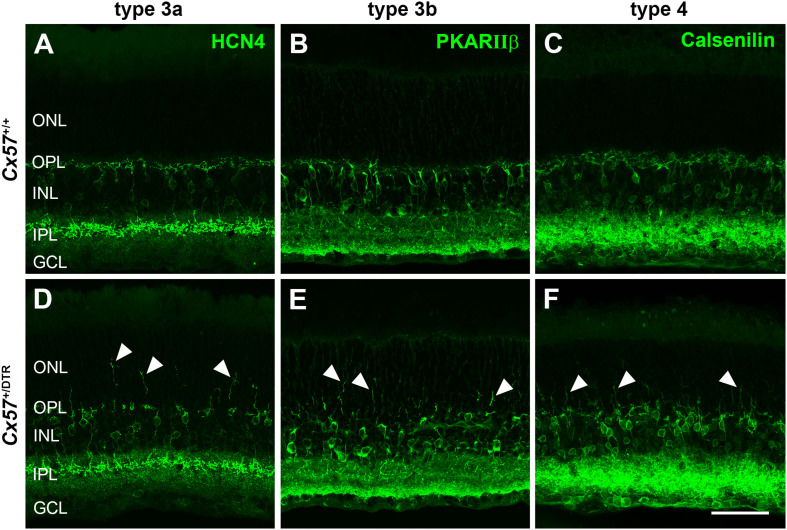
OFF bipolar cells sprouted into the ONL. **(A–F)** Retinal sections from *Cx57*^+/+^ and *Cx57*^+/DTR^ retinae (P56) were stained for HCN4, PKARIIβ and calsenilin, markers for type 3a, 3b, and 4 OFF bipolar cells, respectively. While the dendrites of type 3a, 3b, and 4 OFF bipolar cells terminated in the OPL in wild-type mice **(A–C)**, all three types showed an extensive outgrowth of dendrites into the ONL in horizontal cell-ablated mice (arrowheads) **(D–F)**. Scale bar, 50 μm.

Cone photoreceptors provide synaptic input to five types of OFF bipolar cells (Behrens et al., 2016). To analyze the effects of early postnatal horizontal cell ablation on OFF bipolar cell morphology, we labeled type 3a, 3b, and 4 OFF bipolar cells with antibodies against HCN4, PKARIIβ and calsenilin, respectively. In the adult (P56) wild-type retina, type 3a, 3b, and 4 OFF bipolar cell dendrites exclusively stratified in the OPL (Figures 9A–C). In the horizontal cell-ablated retina, on the contrary, all analyzed types of OFF bipolar cells extended their dendrites into the ONL (Figures 9D–F, arrowheads).

**FIGURE 10:**
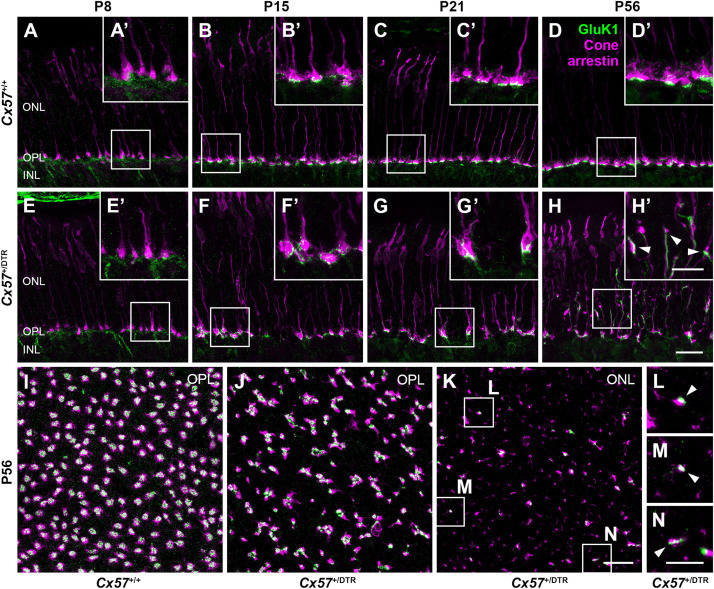
Cones established new synapses with OFF bipolar cells. **(A–H’)** Double labeling of vertical sections from Cx57^+/+^ and Cx57^+/DTR^ retinae for the kainate receptor subunit GluK1 and the cone marker cone arrestin. In wild-type and horizontal cell-ablated mice, GluK1 immunoreactivity was found below the base of the cone terminals in the OPL from P8 to P56 **(A–H)**. In *Cx57*^+/DTR^ retinae, at P56, outgrowing GluK1-positive dendrites made contacts with the newly formed cone terminals in the ONL (arrowheads) **(H’)**. **(I–N)** Double staining of retinal whole mounts from *Cx57^+/+^* **(K)** and *Cx57*^+/DTR^ mice (P56) **(L–N)** for GluK1 and cone arrestin. At the level of the OPL, cone terminals were associated with GluK1 staining in both genotypes **(I,J)**. Ectopic cone terminals in the ONL of horizontal cell-ablated mice were directly connected to GluK1-positive dendrites (arrowheads) **(K–N)**. Scale bars, 20 μm **(H,K)**, 10 μm **(H’,N)**.

Since type 3a, 3b, and 4 OFF bipolar cells not only contact cones but also rods (Mataruga et al., 2007; Haverkamp et al., 2008) and rods have been shown to retract their terminals into the ONL after early postnatal horizontal cell ablation (Nemitz et al., 2019), these OFF bipolar cell types may sprout into the ONL to search for contacts with rods. To test whether outgrowing OFF bipolar cell dendrites in horizontal cell-ablated mice are possibly able to form new synapses with cones, we stained vertical sections and whole mounts for cone arrestin and GluK1, a glutamate receptor subunit that is expressed by type 3a, 3b, and 4 OFF bipolar cells in the mouse retina (Puller et al., 2013). In *Cx57*^+/+^ mice, GluK1 immunoreactivity was present at the base of cone terminals from P8 to P56 (Figures 10A–D’,I), demonstrating that flat contacts with OFF bipolar cells were existing throughout all ages. Similarly, GluK1 staining was found at the base of cone terminals in the OPL of *Cx57*^+/DTR^ mice from P8 onward (Figures 10E–H’,J), suggesting that synapses between cones and OFF bipolar cells are normally formed and retained in horizontal cell-ablated mice. However, in addition to that, we observed several sprouting GluK1-immunoreactive dendrites adjacent to newly formed cone terminals in the ONL at P56 (Figures 10H,H’,K–N). The GluK1 immunoreactivity was often accumulated at ectopic cone terminals (Figures 10H’,L,M), indicating that OFF bipolar cells established new contacts with cones. Since newly formed cone terminals possess ribbons and vesicular glutamate transporters, presynaptic elements that are involved in the glutamate release of photoreceptors, and OFF bipolar cells express postsynaptic glutamate receptors at these terminals, it is highly likely that cones form new synapses with OFF bipolar cells in the adult retina.

## Discussion

The present study aimed to investigate the consequences of early postnatal horizontal cell ablation on the development of cone photoreceptors and their synaptic connections with bipolar cells. Immunohistochemical and electron microscopical analysis revealed that cones initially displayed a normal morphology and formed basal contacts with OFF bipolar cells, but hardly any invaginating contacts with ON bipolar cells. Beginning in the third postnatal week, cones underwent progressive structural and synaptic changes, starting with a loss of synaptic contacts with ON bipolar cells, a mislocalization of cone somata and a clustering of cone terminals. The cone phenotype became even more severe in the adult retina with aberrant neurite sprouting and the establishment of new putative synapses with OFF bipolar cells. These results demonstrate the capacity of the mature retina for structural and synaptic plasticity.

### Loss of Synaptic Contacts Between Cones and ON Bipolar Cells

**FIGURE 11:**
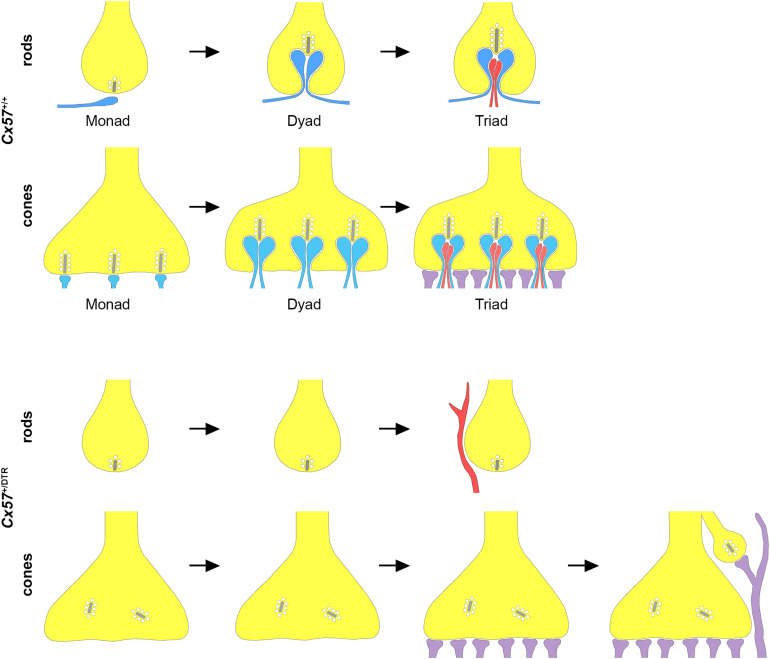
Schematic illustration of rod and cone synaptogenesis in wild-type and horizontal cell-ablated mice. In wild-type mice, the development of rod and cone synapses begins with the formation of a contact between the photoreceptor terminal (yellow) and a single horizontal cell process (blue). Subsequently, a second horizontal cell process is recruited and both horizontal cell processes invaginate into the presynaptic terminal. In the last step, one or two ON bipolar cell dendrites (red) invaginate into the photoreceptor terminal and OFF bipolar cells (violet) form flat contacts at the base of the photoreceptor terminal. In the absence of horizontal cells, ON bipolar cells do not invaginate into rod and cone terminals and rod bipolar cells sprout into the ONL. Compared to wild-type mice, horizontal cell-ablated mice display fewer and shorter presynaptic ribbons, which are often not anchored to the photoreceptor membrane. At later stages, cones form new terminals with synaptic ribbons which are contacted by sprouting OFF bipolar cell dendrites. (Note that synapses between rod terminals and OFF bipolar cells have not been investigated for *Cx57*^+/+^ and *Cx57*^+/DTR^ mice.)

In this study, horizontal cell ablation was induced at the same time as the first contacts between cone terminals and horizontal cells are formed (P4/P5). Therefore, a small number of horizontal cell dendrites was still able to invaginate into cone terminals before horizontal cells were completely eliminated, as evidenced by the presence of some horizontal cell remnants in cone terminals at P11. However, invaginating ON bipolar cell dendrites were never observed without adjacent horizontal cell invaginations at this developmental stage, indicating that cone ON bipolar cells require horizontal cells to invaginate into the cone terminal. In line with this finding, horizontal cells have previously been shown to be essential for the invagination of rod bipolar cells into the rod terminal (Nemitz et al., 2019; Figure 11). From P15 onward, invaginations were completely absent in cone terminals of horizontal cell-ablated mice, suggesting that cones lose all remaining invaginations. This result is consistent with a previous study showing that synaptic contacts between photoreceptors and ON bipolar cells are also lost when horizontal cells are ablated from the adult retina (Sonntag et al., 2012), illustrating that horizontal cells are not only important for the formation, but also for the maintenance of the photoreceptor ribbon synapse. Nevertheless, the molecular mechanism, by which horizontal cells contribute to the formation and maintenance of photoreceptor ribbon synapses remains unresolved. One possibility is that horizontal cells express diffusible or membrane-bound guidance cue molecules that direct the invagination of ON bipolar cells into photoreceptor terminals or stabilize the photoreceptor ribbon synapse. Mouse horizontal cells have been reported to express the cell adhesion molecule NGL-2 (Soto et al., 2013) as well as the semaphorin Sema6a and its receptor PlexA4 (Matsuoka et al., 2012), but the knock-out of *Ngl-2*, *Sema6a* or *PlexA4* does not prevent the invagination of ON bipolar cells into the photoreceptor terminals, indicating that the absence of these molecules is not responsible for the loss of photoreceptor ribbon synapses in horizontal cell-ablated mice. Another possibility is that the synaptic activity of horizontal cells contributes to the formation and maintenance of the photoreceptor ribbon synapse. Previous studies have shown that a loss of photoreceptor proteins that control the release of glutamate leads to a degeneration of photoreceptor ribbon synapses (Dick et al., 2003; Haeseleer et al., 2004; Mansergh et al., 2005; tom Dieck et al., 2012; Michalakis et al., 2013). However, neither the absence of GABA synthesis (Schubert et al., 2010) nor the elimination of the light-dependent modulation of horizontal cell feedback and feedforward (Ströh et al., 2018) alters the ultrastructure of the photoreceptor ribbon synapse, suggesting that the synaptic activity of horizontal cells is not necessary for the assembly and maintenance of photoreceptor ribbon synapses.

### Cone Neurite Sprouting and Formation of New Putative Synapses Between Cones and OFF Bipolar Cells

Aberrant sprouting of photoreceptor neurites has previously been reported for several retinal diseases in humans and animal models of retinal degenerations. Rod photoreceptors show abnormal neurite sprouting in humans with retinitis pigmentosa (Li et al., 1995; Milam and Li, 1996; Fariss et al., 2000), cats with rod/cone dysplasia (Chong et al., 1999), and pigs carrying a mutation in the rhodopsin gene (Li et al., 1998). Furthermore, rods extend beaded axons into the inner retina after retinal detachment (Sethi et al., 2005) and reattachment (Lewis et al., 2002). Cone neurite sprouting has been described for humans with retinitis pigmentosa (Li et al., 1995; Milam and Li, 1996) and age-related macular degeneration (AMD) (Pow and Sullivan, 2007) as well as for two mouse models: *rd*1 (Fei, 2002; Lin et al., 2009) and Ca_*v*_1.4 knock-out mice (Raven et al., 2008; Zabouri and Haverkamp, 2013). *Rd1* mice carry a mutation in the rod cGMP-specific 3′,5′-cyclic phosphodiesterase β subunit gene leading to a primary degeneration of rod photoreceptors, followed by a secondary degeneration of cones and morphological changes in horizontal and bipolar cells (Strettoi and Pignatelli, 2000; Strettoi et al., 2002). In line with our findings, newly formed processes of cones in *rd1* mice often contain synaptic ribbons (Lin et al., 2009). Mice that lack the L-type voltage-dependent calcium channel subunit Ca_*v*_1.4, which is expressed by photoreceptors, display severe structural changes including a loss of invaginations in photoreceptor terminals, abnormal ribbons and dendritic sprouting of second-order neurons (Mansergh et al., 2005; Raven et al., 2008; Zabouri and Haverkamp, 2013). Similar to horizontal cell-ablated mice, cones in Ca_*v*_1.4 mutant mice exhibit an aberrant morphology, showing branched axons and several synaptic terminals (Raven et al., 2008; Zabouri and Haverkamp, 2013). Furthermore, ectopic cone terminals have been shown to establish new synapses with horizontal cells (Zabouri and Haverkamp, 2013). In contrast to that, cone neurite spouting has not been reported for adult *Lim1* conditional knock-out mice in which horizontal cells become misplaced to the inner retina before birth (Poche et al., 2007; Keeley et al., 2013). However, due to incomplete recombination, some horizontal cells remain in the outer retina of *Lim1* conditional knock-out mice which might be sufficient to maintain the normal cone morphology or might induce subtler effects that went undetected. Interestingly, cones do also not display neurite sprouting when horizontal cells are ablated from the adult retina (Sonntag et al., 2012).

What causes cone neurite sprouting after early postnatal horizontal cell ablation? One possibility is that neurite sprouting is a sign for cone degeneration. In favor of this hypothesis are the data presented for *rd1* mice, which showed that neurite sprouting of cones starts at P8 (Fei, 2002) and coincides with the beginning of cone outer segment degeneration in this animal model (Lin et al., 2009). By contrast, in Ca_*v*_1.4 knock-out mice, neurite spouting precedes cone death by several month (Zabouri and Haverkamp, 2013). Moreover, we did not find significant differences in the number of cones at P56 which speaks against this hypothesis. Nevertheless, we cannot rule out that cones die at later stages in horizontal cell-ablated mice. Another reason for cone neurite sprouting after early postnatal horizontal cell ablation may be, that the loss of postsynaptic contacts with horizontal cells and ON bipolar cells triggers the neurite outgrowth and the search for new contacts. Axonal outgrowth and synapse formation rely on extracellular molecular cues that bind to membrane receptors and thereby activate intracellular signaling cascades which result in changes in cytoskeletal dynamics (reviewed in O’Donnell et al., 2009). However, the exact molecular mechanism of axonal outgrowth and synaptogenesis of cones is not yet fully understood. *In vitro* experiments with cultured salamander rod photoreceptors have demonstrated that the guidance cue molecule semaphorin 3A inhibits sprouting of rod photoreceptors (Kung et al., 2017). Murine horizontal cells have been reported to express semaphorin 6A (Sema6A), but in Sema6A knock-out mice, cones do not display aberrant neurite sprouting (Matsuoka et al., 2012), suggesting that the lack of Sema6A is not responsible for cone neurite sprouting in horizontal cell-ablated mice. Moreover, it has been shown that blockage of cGMP-gated channels with cobalt bromide or L-cis diltiazem inhibits neurite outgrowth and varicosity formation whereas activation of cGMP-gated channels with the agonist 8Br-cGMP increases varicosity formation of cones (Zhang and Townes-Anderson, 2002). Furthermore, Pow and Sullivan (2007) observed a strong expression of microtubule associated protein 2 (MAP2), a regulator of neurite outgrowth, in cones of humans with AMD. Whether MAP2 plays a role in cone neurite sprouting after early postnatal horizontal cell ablation remains to be seen. Further investigations will be needed to reveal the precise mechanisms underlying axonal outgrowth and synaptogenesis of cones in horizontal cell-ablated mice. Nevertheless, our study underlines the potential of the mature retina for morphological plasticity and provides the first evidence that adult cones may be able to form new synapses with OFF bipolar cells.

## Data Availability Statement

The original contributions presented in the study are included in the article/Supplementary Material, further inquiries can be directed to the corresponding author/s.

## Ethics Statement

The animal study was reviewed and approved by Niedersächsisches Landesamt für Verbraucherschutz und Lebensmittelsicherheit.

## Author Contributions

LN, KD, and UJ-B designed the experiments and contributed to the interpretation of data. LN and UJ-B performed the experiments. LN prepared the figures and wrote a first draft of the manuscript. KD and UJ-B revised the manuscript. All authors contributed to the article and approved the submitted version.

## Conflict of Interest

The authors declare that the research was conducted in the absence of any commercial or financial relationships that could be construed as a potential conflict of interest.
