# Supplementary material for: Synaptic Remodeling in the Cone Pathway After Early Postnatal Horizontal Cell Ablation
Source: Front Cell Neurosci. 2021 May 26;15:657594. doi: 10.3389/fncel.2021.657594 (PMC8187617; doi:10.3389/fncel.2021.657594)
Supplement: Supplementary file 1 [file Table_1.docx]

Supplementary Material

# Supplementary Data

# Supplementary Figures

## Supplementary Figures


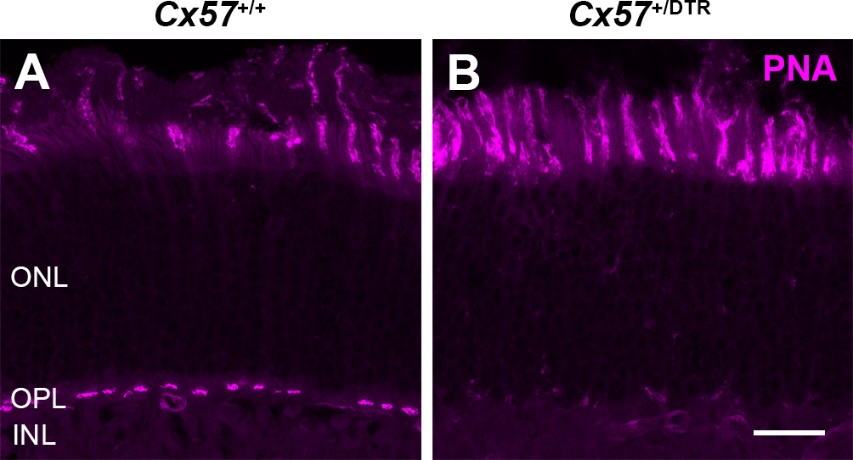


**Supplementary Figure 1.** **PNA labeling in the OPL is reduced after early postnatal horizontal cell ablation.** **(A-B)** Vertical cryosections of *Cx57*^+/+^ and *Cx57*^+/DTR^ retinae were labeled with PNA conjugated to Alexa 568 (Thermo Fisher Scientific, Catalog #: L32458). Compared to wild-type mice, PNA staining in the OPL is strongly decreased in horizontal cell-ablated mice. Scale bar, 50 µm.
